# Neuropsychological Performance in Polyconsumer Men Under Treatment. Influence of Age of Onset of Substance Use

**DOI:** 10.1038/srep12038

**Published:** 2015-07-09

**Authors:** Maria del Mar Capella, Irina Benaiges, Ana Adan

**Affiliations:** 1 Department of Psychiatry and Clinical Psychobiology, University of Barcelona, Passeig Vall d’Hebrón 171, 08035 Barcelona, Spain; 2 Institute for Brain, Cognition and Behavior (IR3C), Barcelona, Spain

## Abstract

Neurocognition is a key factor in the development and maintenance of Substance Use Disorders (SUD). However, there are still several aspects that need to be studied in this area. In this study, we elucidate the influence of age of onset of substance use (OSU) on the clinical course and neuropsychological performance of substance use disorder (SUD) patients, as well as to explore the influence of years of education, duration of drug use and premorbid intelligence quotient (IQ) on the cognitive results obtained. An exhaustive neuropsychological battery was used to assess different cognitive domains in 80 male polyconsumers, 41 with earlier OSU (16 years or before: OSU ≤ 16) and 39 with later OSU (17 years or later: OSU ≥ 17). The patients were under treatment with at least 4 months of abstinence confirmed by urinalysis. The OSU ≤ 16 group presented a worse clinical state, as well as a lower premorbid IQ and worse performance in processing speed, visual perception and planning skills. The duration of drug use may account for the differences in planning and processing speed. In this work we discuss the premorbid or acquired nature of the cognitive deficits found.

The United Nations Office on Drugs and Crime considers substance use as a public health issue that has severe consequences on individuals and communities^1^. Despite the improvements in prevention and treatment of Substance Use Disorders (SUD), the world levels of consumption are significantly high^2^. This may be partly due to the fact that several genetic and environmental factors intervene in the onset and maintenance of SUD^3^, which in turn causes a wide array of clinical symptomatology^4^ and response to intervention^5^. Thus, it is necessary to study new ways to improve our knowledge of the ethiopathogenesis of SUD, its typologies and the relevant associated variables both its prevention and rehabilitation.

In this line, neuropsychology has made valuable contributions in recent years. Some studies, scarce but promising, indicate that incorporating cognitive rehabilitation in the treatment of addiction optimizes the results of traditional interventions^6^,^7^. This is consistent with current models of development and maintenance of SUD where the role of biological and neurocognitive factors stands, in addition to environmental factors^3^.

Thus, it has been shown that SUD patients show alterations in cognitive functions such as inhibitory or executive control^8^,^9^, working memory^10^ or visuospatial skills^11^, even before consumption onset. In addition, several studies support that cognitive impairment due to consumption is related to both duration and severity of addiction, there being dose-dependent relationships^12^, although more studies are needed to establish a reliable relationship between cognitive impairment and the severity of SUD. Likewise, it has been shown that the greater the cognitive impairment, poorer are the treatment outcomes^13^,^14^. Specifically, deficits in inhibitory control and cognitive flexibility may affect the ability of patients to focus attention and direct their behavior to new and alternative goals which are incompatible with substance use-related behaviors^15^.

In the study of the relationship between cognitive functions, and the development and characteristics of SUD, special attention has been paid to modulating factors such as the type of substance used^16^, the pattern of consumption or duration of drug use^17^. While some studies have also highlighted the relevance of the age of onset of substance use (OSU), new studies are needed to provide more robust data about their relationship, particularly in cases of polyconsumption or considering specific ages of OSU. However, in samples of alcoholic patients, an earlier OSU has been linked to increased intensity of personality traits linked to consumption, such as high Novelty Seeking or low Harm Avoidance^6^,^18^, worse clinical course^4^ and greater structural and functional brain alterations^19^. In polyconsumers, early OSU has also been associated with greater cognitive impairment^20^. Furthermore, consumption in adolescence implies greater cognitive impairment compared to adulthood^21^,^22^, due to the different critical periods for brain maturation^23^. Thus, the study of typologies of addicts based on their cognitive performance and their clinical implications considering the age of OSU is a research area of undoubted clinical interest.

In several studies with cannabis consumers, neuropsychological differences have been observed depending on whether consumption begins at age 16 or earlier, or at age 17 or later. Individuals with an earlier OSU perform lower in tasks of visual exploration^24^, processing speed and cognitive flexibility^25^; have lower verbal intelligence quotient (VIQ)^26^ and show less cerebral and gray matter volume^27^. The cut-off age used in these studies was based on three characteristics of brain ontogeny: (a) between 12 and 15 years the network involved in visual scanning reaches its peak development^28^; (b) compared with the serotonergic system, the dopaminergic and endocannabinoid systems, which are key in prefrontal functioning, mature earlier^29^, being almost defined by the end of puberty^30^; (c) by age 15 there is one last peak in cortical changes^31^. However, the only study that used this cutoff age with mostly cocaine and alcohol consumers did not replicate earlier findings^32^.

Our work has three aims. The first is to study the differences in the clinical course of polyconsuming men diagnosed with SUD, depending on whether they initiated substance use at age 16 or earlier (OSU ≤ 16) or at age 17 or later (OSU ≥ 17). The second is to assess the differences in their neuropsychological performance. Unlike previous works where only one type of cognitive function has been studied, in the current work we have administered a comprehensive battery of neuropsychological tests sensitive to the characteristics of SUD patients. Our third goal is to explore, regardless of the groups, if the age of patients, age of OSU, years of education, duration of drug use and the premorbid intelligence quotient (IQ) modulate their neurocognitive performance.

## Results

### Differences in sociodemographic and clinical data

With respect to sociodemographic variables, the OSU ≤ 16 and OSU ≥ 17 groups provided no significant differences in any of them: age, years of education, marital and economic status. The overall sample was aged 20 to 55 (36.45 ± 8.20) and most of the patients had completed the Spanish compulsory education (from 6 to 16 years, grades 1 to 10). The analyses of the clinical variables did indicate significant differences between groups, being more frequent in the OSU ≤ 16 group to have relatives with SUD (p = 0.044). See Table 1.

Regarding SUD data, the OSU ≤ 16 group had a higher rate of patients in residential rather than in ambulatory treatment (p = 0.003) and with a higher rate of polyconsumption (p = 0.008). They also had lower age of OSU (p = 0.0001), greater duration of drug use (p = 0.001) and higher rates of relapse (p = 0.039). The groups did not differ in the type of substance used or in the months of abstinence (see Table 2). In the total sample, the substances most frequently used were cocaine (95%), alcohol (77.5%) and cannabis (48.8%).

In the total sample, an additional analysis was carried out considering the treatment regimen (residential or ambulatory), to assess whether this was an indicator of clinical severity related to the recruitment of patients in both regimes and not related to the age of OSU. No significant differences between groups were found regarding age (*t*_(39)_ = 1.899; p = 0.065), years of education (*U* = 549.50; p = 0.102) or scores in the Block Design subtest (*t*_(39)_ = 1.19; p = 0.544). Instead, significant differences were found in duration of drug use (*t*_(39)_ = 0.826; p = 0.030) and in the Vocabulary subtest scores (*t*_(39)_ = −0.764; p = 0.016).

### Differences in neuropsychological functioning

Considering age and years of education as covariates, significant differences between groups were found in the Vocabulary (p = 0.007) and Block Design (p = 0.019) subtests, where the OSU ≤ 16 group had a worse performance in both. This group also took longer (p = 0.003) to complete the Trail Making Test part A (TMT-A). Regarding the Judgment of Line Orientation Test (JLOT), the OSU ≤ 16 group performed worse in all the parameters measured: number of correct answers (p = 0.024) and reaction time (p = 0.038) (see Table 3).

In addition, in the Tower of Hanoi test the OSU ≤ 16 group required more number of movements (p = 0.045), committed more errors (p = 0.262) and showed a higher reaction time (p = 0.009) (see Table 4). No differences between groups were found in the other neuropsychological tasks: Digits Forward, Digits Backwards, Rey Auditory Verbal Learning Test (RAVLT), Trail Making Test part-B (TMT-B), Wisconsin Card Sorting Test (WCST) and Iowa Gambling Test (IGT).

### Influence on neuropsychological functioning of age of onset of substance use, age, years of education, duration of drug use, premorbid verbal and performance IQ

First, comparing cognitive performance taking age, years of education, duration of drug use and score in Vocabulary and Block Design as covariates eliminated intergroup differences observed previously (see Table 3 and Table 4).

Second, considering the total sample, the regression analysis indicated that the model was significant in only three neuropsychological tasks: TMT-A, JLOT and Tower of Hanoi (see Table 5). In these three cognitive domains, the significant variables were Block Design, duration of drug use and age of OSU, explaining more than 17% of the variance. Block Design explained 34% of the variance of the number of correct answers in the JLOT (p = 0.0001) and, together with age of OSU, 17% of the variance of reaction time (p = 0.0001). Block Design and duration of drug use accounted for 29% of the variance in the TMT-A. Finally, duration of drug use explained 20% of the variance in reaction time in the Tower of Hanoi (p = 0.0001).

## Discussion

This study examines, for the first time, the possible existence of clinical and neurocognitive differences in polydrug addicts depending on whether their substance use began at age 16 or earlier, or at age 17 or later. In addition, we also assess the effect of age of onset, age, years of education, duration of drug use and premorbid IQ on cognitive performance for the total sample.

The groups did not differ in any sociodemographic parameter studied or in the main substance of consumption. Moreover, they did not differ in the variables that affect cognition, such as duration of abstinence^33^,^34^, rates of caffeine and nicotine intake^35^ or use of psychotropic drugs^36^, and therefore we discarded their effects on the results of neuropsychological performance.

The OSU ≤ 16 group presented a more severe clinical pattern, characterized by the presence of more family history of SUD, higher relapse rate, the need for a more intensive treatment regimen to achieve abstinence, greater duration of drug use and consumption of more substances. Only Pope *et al.*^26^ studied the family history of substance abuse in cannabis consumers, obtaining no differences between the OSU ≤ 16 and OSU ≥ 17 groups. However, our results on the age of OSU are revealing and link with the observation that, for alcoholic patients, younger ages of first use have been associated with worse clinical course of SUD^4^.

We observed several differences in neuropsychological functioning between groups. Controlling the effect of age and years of education, the OSU ≤ 16 group presented lower premorbid IQ in both the verbal component (VIQ), measured using the Vocabulary subtest and in the performance component (PIQ), measured through the Block Design subtest. These cognitive differences have not been found in cannabis addicts with brief periods of abstinence^24^. In contrast, consumers with age of OSU ≤ 16 and longer periods of abstinence, more similar to our sample, show lower VIQ^26^. This suggests that when patients begin consumption at age 17 or later, with the maintenance of abstinence the speed of recovery in global cognitive ability is higher than in those who began consumption at age 16 or earlier.

Two hypotheses might explain the lower IQ of the OSU ≤ 16 group: the existence of a worse cognition prior to consumption or a higher effect of overall neurotoxicity associated to the age of OSU. In the case of lower VIQ, some results seem to be more indicative of the hypothesis of lower cognitive ability prior to consumption: (a) participants in this group had a higher rate of family history of SUD, a characteristic related to the presence of cognitive impairment in their descendants even if the latter have not consumed^29^; (b) the estimate is made from the Vocabulary subtest, where performance is more preserved after neurological damage, thus making it the most widely accepted as a measure of premorbid cognitive functioning^35^. In contrast, the origin of the low PIQ raises more questions. In this case, other evidence supports the hypothesis of a greater brain damage arising from SUD: (a) although the Block Design subtest is commonly used as a measure of premorbid PIQ, its performance is sensitive to attacks to the central nervous system^37^; (b) since this task is related to perceptual organization^35^, whose neurological substrate shows a critical maturation period prior to age 16^38^, consumption at age 16 or earlier could promote aberrant synaptic reorganizations that would chronically alter its functionality. However, it is noteworthy that, contrary to this second hypothesis, visuospatial deficits have been found in patients with a family history of SUD^11^ as well as lower PIQ associated with development of SUD in adulthood^39^, which would support the hypothesis of a worse premorbid PIQ. Unfortunately, the design of our study does not allow us to clarify these issues at the moment.

In addition, the OSU ≤ 16 group showed lower speed of processing and slower visuoperceptual skills, as well as higher deficits in planning. However, no differences were observed in tasks of attention, verbal memory, working memory, cognitive flexibility or abstract reasoning, nor were there alterations in the processes of risk decision-making. Moreover, when premorbid IQ and duration of drug use were controlled in the analyses, all cognitive differences between groups disappeared. The linear regression analyses with the total sample allowed us to elucidate these results.

At lower PIQ scores, the patients performed worse in the task of visual perception and showed slower processing speed. Therefore, we cannot confirm that consumption at age 16 or earlier is related to higher deficits in these skills since the lower premorbid IQ interferes with their performance.

However, it should be highlighted that the age of OSU also modulated the visuoperceptual performance of the participants: when it was lower, they needed more time to respond to the stimuli in this task. This would support the idea that drug consumption at age 16 or earlier may alter the optimal neurodevelopment, and its consequence would imply specific visuoperceptual deficits. Since in our area of study no previous work had considered this cognitive function^24^,^25^,^26^,^32^, more data are needed to sustain this hypothesis.

Nevertheless, our results indicate that the variable with greater explanatory power regarding planning abilities and processing speed is the duration of drug use. This is consistent with evidence that repeated use of substances is associated with morphological brain changes in both gray and white matter. In polydrug patients, as consumption becomes chronic, it has been observed greater volume reduction in the prefrontal cortex^40^, associated with planning skills^41^. In cocaine users, increased abnormalities in the corpus callosum also correlate with greater impulsivity and lack of planning^42^,^43^. Therefore, differences in duration of drug use could underlie some results that, unlike ours, have observed different executive functioning and processing speed between the OSU ≤ 16 and OSU ≥ 17 groups^25^.

The interpretation of our results is subject to limitations. Most of the sample consisted of polydrug users, making it impossible to separate the differential effect of each type of substance on neurocognition^16^. Although this is a frequent limitation with this type of patients^44^, in our study its effect is relatively controlled since the groups did not differ in the main substances used. Future studies should considered the main substance associated with the diagnosis of dependence, since it is possible that it plays a role in the explanatory model of performance in visual perception, planning and processing speed, together with the age of OSU, duration of drug use and PIQ. In addition, it would be interesting to incorporate designs that contribute to clarify the etiology and clinical course of cognitive deficits observed in the OSU ≤ 16 group and its relation to clinical severity.

Although our results should be interpreted with caution, they may have clinical implications. SUD patients may benefit from cognitive rehabilitation^6^,^7^, because cognitive deficits and clinical course are related^12^. Therefore, neuropsychological assessment and rehabilitation in SUD treatment programs, especially in patients with OSU ≤ 16, could improve adherence and response to interventions. This is relevant because our results confirm that the duration of drug use is related to cognitive impairment on executive performance and processing speed, which could increase the difficulty of rehabilitation^15^. Finally, it would be interesting to develop longitudinal studies assessing the presence of cognitive disorders in the OSU ≤ 16 group prior to consumption and, therefore, help to consider this age group as a target population in primary prevention programs.

In conclusion, the cut-off age considered in our study allows us to differentiate typologies of polydrug addicts in relation to their clinical severity and cognitive functioning, so that taking them into consideration could contribute to improve SUD prevention and treatment programs. The OSU ≤ 16 group presents a more severe clinical pattern: higher rates of family history of SUD, greater number of relapses, a consumption pattern characterized by the use of more substances and the need for interment to achieve similar abstinence. They also show lower IQ scores, higher visuoperceptual and planning deficits and slower processing speed. The lower VIQ could be a premorbid characteristic, and the lower PIQ and visuoperceptual skills could be either the result of consumption on the neurodevelopment as characteristics prior to the SUD. Further work is required to shed light on this issue. The difficulties in planning and greater slowdown in information processing may be related to the duration of drug use, which make it highly relevant both for the study of neuropsychological characteristics in the field of addictions as well as for tertiary prevention programs.

## Methods

### Participants

In a cross-sectional study design, we enrolled 80 patients linked to different healthcare resources for SUD treatment. All were male, given the high prevalence of this gender in admissions to treatment for SUD^45^ and to avoiding bias on the results due to sex differences^46^. Informed consent was obtained from all participants, who were not compensated for their collaboration in the study.

The inclusion criteria were: current or past diagnosis of SUD, ongoing treatment, confirmed by a diagnostic interview according to Diagnostic and Statistical Manual of Mental Disorders, Fourth Edition Text Revised (DSM-IV-TR)^47^ criteria, established by their treating clinician or a trained clinical researcher; with abstinence for at least 4 months at the time of the study (excluding caffeine or nicotine consumption), confirmed by urinalysis. The exclusion criteria were: female gender; age below 18 and above 55; presence of mental retardation or pervasive developmental disorder, history of traumatic brain injury or neurological injury, or cognitive or physical impairment that would preclude the correct application of the selected tests; and presence of a comorbid axis I mental disorder confirmed by a diagnostic interview according to DSM-IV-TR criteria.

The patients were added consecutively according to the centers’ referral, without taking into account the OSU variable. After collecting the information, the sample was classified into two groups depending on whether substance use had begun at age 16 or earlier (OSU ≤ 16; n = 41) or at age 17 or later (OSU ≥ 17; n = 39). The age cut-off was based on neurodevelopmental characteristics, similarly to what had been done in previous studies with cannabis consumers^28^,^29^,^30^,^31^.

Experimental protocol of this study was approved by the University of Barcelona’ ethic committee and the methods were carried out in accordance with the ethical principles of the declaration of Helsinki.

### Clinical measures

Information was collected on sociodemographic (age, marital status, educational and economic status) and clinical (presence of organic pathology, psychiatric and substance use family history, suicidal attempts, past treatment for SUD, consumption pattern, type of drugs used, age of onset substance use, duration of drug use, typology of treatment regimen, medication, abstinence periods and relapses) variables, using a structured interview. This information was confirmed with the medical history of the centers database and with the patients treating psychiatrist. Furthermore, daily consumption of cigarettes and caffeine beverages was recorded. The *Fagerström* test of nicotine dependence^48^ was administered to smokers. All participants were administered the Clinical Global Impression questionnaire (CGI)^49^, as a subjective measure of the clinical severity.

### Neuropsychological assessment

Cognitive functioning was assessed by a comprehensive battery of cognitive measures, extensively validated and routinely used^35^. The administration of the tests was distributed into two separate sessions of two hours each, always in a fixed order alternating verbal and manipulative tests. All participants completed the battery. The Vocabulary and Block Design subtests of the Wechsler Adult Intelligence Third Edition Scale Revised (WAIS-III)^50^ were administered to assess the premorbid VIQ and PIQ, respectively^35^. Attention span was measured with the Digit Span Subtest of the WAIS-III. The TMT-A^51^ was administered as a measure of processing speed. We assessed visuospatial perception with the JLOT^52^, in there computerized version of Estévez-González (2001). Declarative, immediate and delayed memory variables were assessed with the RAVLT^53^. Finally, we measured the performance in different components of executive functions: the TMT-B^51^ and the Digits Backwards Subtest of the WAIS-III for the working memory; the Tower of Hanoi^54^, in there four disk computerized version of González-Vilches (2000) for the planning and problem solving; the WCST^55^, in there computerized version of Estévez-González (2001), for the cognitive flexibility, reasoning and problem solving; and the IGT^56^, in there computerized version of Kilgard (1997), for the decision making with risk.

### Statistical analysis

Differences between groups in the sociodemographic and clinical variables were explored with the Mann-Whitney U test *(U)* or with the Chi Square test for categorical variables. If the quantitative data fulfilled the necessary conditions, the Student’s t-test *(t)* was used; when the conditions were not met, the nonparametric Mann-Whitney U test *(U)* was used instead.

Differences in neuropsychological performance were assessed using analysis of covariance (ANCOVA), multiple analysis of covariance (MANCOVA) or with repeated measures MANCOVA (RM MANCOVA), depending on the task. The Bonferroni test was applied in all analyses to reduce the occurrence of a type 1 error. The effect size was calculated with the partial Eta squared *(η*_*p*_^2^) index, assuming a value of 0.01 as low, of 0.04 as moderate and of 0.1 as high^57^. Age and years of education were considered as covariates, given their known effects on cognitive performance^58^,^59^ and because they presented high standard deviations in both groups.

The two groups differed in duration of drug use and in the scores obtained in the Vocabulary and Block Design subtests of the Wechsler Adult Intelligence Scale-Revised Third Edition (WAIS-III). These subtests are considered measures of premorbid IQ^35^,^60^. Moreover, the effects of duration of drug use on neuropsychological performance are well known^35^,^44^. Thus, we explored their possible influence on the neuropsychological results, together with age and years of schooling. This was done in two steps. Firstly, the analyses of covariance were repeated for all the cognitive tasks considering the five covariates. Secondly, a confirmatory analysis was performed using a stepwise regression analysis, in order to study their influence on those neuropsychological tasks in which a loss of significance was observed when compared to the first analysis. We consider the total sample, considering the neuropsychological measures as dependent variables and introducing as independent variables the age of OSU, age, years of schooling and duration of consumption, and the scores in the Vocabulary and Block Design subtests. Data were analyzed using the Statistical Package for the Social Sciences (SPSS; version 15.0), considering bilateral statistical significance with an established type 1 error at 5% (p < 0.05).

## Additional Information

**How to cite this article**: Capella, M. M. *et al.* Neuropsychological Performance in Polyconsumer Men Under Treatment. Influence of Age of Onset of Substance Use. *Sci. Rep.* **5**, 12038; doi: 10.1038/srep12038 (2015).

## Figures and Tables

**Table 1:** Descriptive statistics (frequencies or mean and standard error) of the sociodemographic and clinical data for the two groups and the statistical contrasts carried out.

	OSU ≤ 16 (N = 41)	OSU ≥ 17 (N = 39)	Statistical contrasts	*p* values
Sociodemographic data				
Age (yr)	34.83 ± 1.29	38.15 ± 1.26	*t* = −1.84	0.070
Years of education	10.02 ± 0.29	10.72 ± 0.43	*U* = 634.50	0.097
Marital status			*X*^2^ = 2.49	0.646
Single	56.1%	51.3%		
Stable partner	9.8%	5.1%		
Married	19.5%	17.9%		
Separate/Divorced	14.6%	25.6%		
Economic status			*X*^2^ = 4.47	0.346
Active	22.0%	43.6%		
Disability pension	24.4%	15.4%		
Sick leave	4.9%	2.6%		
Unemployed	26.8%	20.5%		
No income	22%	17.9%		
Clinical data				
Relatives with SUD			*U* = 666.00	0.044
Yes	24.4%	7.7%		
No	75.6%	92,3%		
Relatives with psychiatric disorder			*U* = 772.50	0.762
Yes	65.9%	69.2%		
No	34,1%	30.8%		
Number of suicidal attempts	0.39 ± 0.14	0.13 ± 0.05	*U* = 716.50	0.227
Past treatment for SUD			*U* = 773.50	0.762
Yes	36.4%	33.3%		
No	63.4%	66.7%		
Daily number of cigarettes for tobacco smokers	13.05 ± 1.12	12.38 ± 1.5	*U* = 760.00	0.698
Fagerström Score for tobacco smokers			*U* = 483.00	0.913
Low Dependence	34.3%	32.1%		
Moderate Dependence	54.3%	57.1%		
High Dependence	11%	10.7%		
Daily beverages with caffeine	2.23 ± 0.28	3.78 ± 1.24	*U* = 679.50	0.238

OSU ≤ 16: Onset of substance use at age 16 or earlier; OSU ≥ 17: Onset of substance use at age 17 or later; yr: years; SUD: Substance Use Disorder.

**Table 2:** Descriptive statistics (frequencies or mean and standard error) of data related to SUD for the two groups and the statistical contrasts carried out.

Substance use data	OSU ≤ 16 (N = 41)	OSU ≥ 17 (N = 39)	Statistical contrasts	*p* values
Consumption pattern				
One substance	12.2%	23.1%	*U* = 712.50	0.203
Two substances	22.1%	41.0%	*U* = 647.00	0.068
Polydrug use	65.7%	35.9%	*U* = 560.00	0.008
Substances used^a^				
Cocaine	95.1%	94.9%	*U* = 797.50	0.959
Alcohol	82.9%	71.8%	*U* = 710.50	0.236
Cannabis	58.5%	38.5%	*U* = 639.00	0.074
Opioids	22%	15.4%	*U* = 747.00	0.455
Ecstasy	22%	7.7%	*U* = 685.50	0.076
Hallucinogens	17.1%	5.1%	*U* = 704.00	0.093
Sedatives	4.9%	5.1%	*U* = 797.50	0.959
Age of OSU (yr)	14.93 ± 0.19	23.03 ± 1.12	*U* = 1.50	0.0001
Duration of drug use (yr)	19.39 ± 1.26	13.12 ± 1.62	*t* = 3.58	0.001
Typology of treatment regimen			*X*^2^ = 9.12	0.003
Residential	82.9%	51.3%		
Ambulatory	17.1%	48.7%		
Number of psychotropics including treatment			*U* = 689.50	0.215
None	56.1%	71.8%		
One	26.8%	12.8%		
More than one	17.1%	15.4%		
Months of abstinence	7.76 ± 0.71	8.31 ± 0.73	*U* = 713.00	0.399
Number of relapses	1.49 ± 0.32	0.64 ± 0.16	*U* = 679.50	0.039
Clinical Global Impression (CGI)	2.36 ± 0.23	2.16 ± 0.20	*U* = 681.00	0.659

^a^Percentages will not equal 100 as each participant may take more than one substance of abuse.

OSU ≤ 16: Onset of substance use at age 16 or earlier; OSU ≥ 17: Onset of substance use at age 17 or later; OSU: Onset of substance use; yr: years.

**Table 3:** Results of ANCOVA, MANCOVA or RM MANCOVA analyses considering two or five covariables, with mean and standard deviations of the groups of patients in a first group of neuropsychological tasks.

Neuropsychological tasks	OSU ≤ 16 (N = 41)	OSU ≥ 17 (N = 39)	Age and Years of education covariables			Age, Years of education, Duration of drug use (yr), Vocabulary and Block Design Scores covariables		
			F	*p* values	Effect Size	F	*p*values	Effect Size
PREMORBID IQ								
Vocabulary (WAIS-III). Direct Score	40.24 ± 6.76	44.97 ± 5.94	7.71	0.007	0.09			
Block Design (WAIS-III). Direct Score	41.09 ± 11.56	46.31 ± 10.06	5.74	0.019	0.07			
ATTENTION SPAN								
Digits Forward (WAIS-III). Direct Score	8.10 ± 1.63	8.77 ± 1.88	1.52	0.221	0.02	0.40	0.530	0.10
PROCESSING SPEED								
TMT-A (Seconds)	27.15 ± 8.84	22.28 ± 7.04	9.42	0.003	0.11	0.99	0.322	0.17
VISUOSPATIAL PERCEPTION								
Judgment of Line Orientation Test								
Number of correct answers	23.60 ± 3.90	25.44 ± 3.19	5.30	0.024	0.07	0.12	0.726	0.64
Reaction Time (milliseconds)	7487.18 ± 2576.25	6455.85 ± 2044.21	4.47	0.038	0.06	0.05	0.822	0.05
VERBAL MEMORY								
AVLT (Number of recorded words)			0.40^a^	0.531	0.01	0.34^a^	0.847	0.13
A1	5.24 ± 1.32	5.82 ± 1.60	1.67	0.201	0.02	0.87	0.355	0.15
A2	8.12 ± 1.82	8.15 ± 1.84	0.00	0.977	0.00	0.49	0.488	0.11
A3	9.93 ± 2.01	10.13 ± 2.52	0.11	0.739	0.00	0.31	0.577	0.09
A4	11.02 ± 1.98	11.23 ± 2.21	0.08	0.775	0.00	0.08	0.775	0.06
A5	11.80 ± 2.21	12.21 ± 1.92	0.82	0.367	0.00	0.16	0.687	0.07
Total words from list A	46.10 ± 8.09	47.54 ± 8.47	0.41	0.523	0.01	0.00	0.994	0.05
B1 (interference list)	4.98 ± 1.33	5.00 ± 1.70	0.00	0.955	0.00	0.13	0.720	0.07
A6	9.27 ± 2.96	9.74 ± 2.82	0.58	0.448	0.01	0.71	0.403	0.13
A7	9.07 ± 2.90	9.38 ± 2.73	0.41	0.522	0.01	0.10	0.753	0.06
REC A/15	13.49 ± 1.69	13.35 ± 1.66	0.20	0.656	0.00	0.39	0.536	0.09

^a^Results of the RM MANCOVA for five trials.

yr: years; OSU ≤ 16: Onset of substance use at age 16 or earlier; OSU ≥ 17: Onset of substance use at age 17 or later; IQ: intelligence quotient;WAIS-III: Wechsler Adult Intelligence Scale-Revised Third Edition; TMT-A: Trail Making Test part A; AVLT: Auditory Verbal Learning Test; A1, A2, A3, A4, A5: Number of words recalled in 5 consecutive trials from list A; B1: Number of words recalled from list B; A6: Number of words recalled from list A immediately after the recall list B; A7: Delayed recall of List A after 15 minutes; REC A/15: Number of correctly recognized words from list A.

**Table 4:** Results of ANCOVA or MANCOVA analyses considering two or five covariables, with mean and standard deviations of the groups of patients on executive function tests.

Executive functions tests	OSU ≤ 16 (N = 41)	OSU ≥ 17 (N = 39)	Age and Years of education covariables			Age, Years of education, Duration of drug use (yr), Vocabulary and Block Design Scores covariables		
			F	*p* values	Effect Size	F	*p*values	Effect Size
Digits Backwards (WAIS-III). Direct Score	5.88 ± 2.00	6.33 ± 1.97	0.62	0.433	0.01	0.01	0.975	0.05
TMT-B (seconds)	73.20 ± 33.0	67.51 ± 36.48	0.96	0.331	0.01	0.24	0.623	0.08
Tower of Hanoi								
Number of movements	29.95 ± 11.71	24.95 ± 9.26	3.90	0.045	0.05	1.56	0.217	0.23
Number of errors	1.68 ± 2.00	1.34 ± 2.17	1.27	0.262	0.02	0.21	0.651	0.07
Reaction Time (seconds)	209.38 ± 145.90	149.97 ± 78.95	7.22	0.009	0.09	0.14	0.712	0.07
WCST								
Trials administered	91.23 ± 18.22	91.10 ± 18.41	0.10	0.752	0.00	1.71	0.195	0.25
Total correct	73.95 ± 11.79	72.44 ± 8.60	0.03	0.870	0.00	0.68	0.412	0.13
Total errors (%)	17.35 ± 8.58	18.74 ± 7.33	0.63	0.429	0.01	1.51	0.223	0.29
Perseverative errors (%)	4.80 ± 7.09	5.08 ± 4.86	0.00	0.952	0.00	1.00	0.321	0.18
Non-perseverative errors (%)	11.35 ± 3.27	15.23 ± 12.24	3.49	0.066	0.04	0.18	0.666	0.07
Conceptual level responses (%)	76.23 ± 15.72	76.41 ± 10.74	0.06	0.806	0.00	0.62	0.434	0.12
Categories completed	5.85 ± 0.80	5.72 ± 1.08	0.35	0.554	0.01	0.41	0.522	0.10
Trials to first category	14.65 ± 13.20	15.72 ± 18.83	0.04	0.853	0.00	1.30	0.258	0.20
Failure to maintain set	0.90 ± 1.26	0.95 ± 1.52	0.18	0.693	0.00	0.76	0.387	0.14
Learn to learn	−1.68 ± 4.29	−1.15 ± 4.45	0.37	0.548	0.01	0.02	0.889	0.05
Reaction Time (milliseconds)	3307.65 ± 1256.26	2950.62 ± 1324.49	1.31	0.256	0.02	0.04	0.836	0.06
Iowa Gambling Task								
Total of the 100 trials	1.84 ± 20.82	12.92 ± 31.52	2.15	0.147	0.03	1.92	0.171	0.28

OSU ≤ 16: Onset of substance use at age 16 or earlier; OSU ≥ 17: Onset of substance use at age 17 or later; WAIS-III: Wechsler Adult Intelligence Scale-Revised Third Edition; yr: years. TMT-B: Trail Making Test part B; WCST: Wisconsin Card Sorting Test.

**Table 5:** Multiple linear regression for each neuropsychological task that has shown differences between groups considering the independent variables Age of onset of substance use, Age, Years of education, Duration of drug use, Vocabulary and Block Design Scores; for the total sample.

Neuropsychological tasks	Adjusted R	F	IV^a^	β Standardized	*p* values	Tolerance	VIF
TMT-A (seconds)	0.29	16.84	Duration of drug use (yr)	−0.42	0.0001	0.91	1.09
			Block Design (Direct Score)	0.26	0.02	0.91	1.09
Judgment of Line Orientation Test							
Number of correct answers	0.34	41.72	Block Design (Direct Score)	0.59	0.0001	1.00	1.00
Reaction Time (milliseconds)	0.17	9.22	Age of onset of substance use (yr)	−0.26	0.01	0.99	1.01
			Block Design (Direct Score)	−0.33	0.002	0.99	1.01
Tower of Hanoi							
Number of movements^b^							
Reaction Time (seconds)	0.20	19.12	Duration of drug use (yr)	0.46	0.0001	1.0	1.0

^a^Only significant variables are presented that comprise each explicative model.

^b^Any explicative model was significant.

IV: Independent Variables; VIF: Variance inflation factor; TMT-A: Trail Making Test part A; yr: years.
